# Computer Vision in Lower Limb Orthopaedics: A Scoping Review of Imaging-Based Artificial Intelligence Applications

**DOI:** 10.7759/cureus.98648

**Published:** 2025-12-07

**Authors:** Nimra Akram, Sirtaaj Mattoo, Bilal Qaddoura, Donia Karimaghaei, Islam Hamarsheh, Sarkhell Radha

**Affiliations:** 1 Trauma and Orthopaedics, Royal Berkshire NHS Foundation Trust, Reading, GBR; 2 Trauma and Orthopaedics, University Hospital Southampton NHS Foundation Trust, Southampton, GBR; 3 Trauma and Orthopaedics, Epsom and St Helier University Hospitals NHS Trust, London, GBR; 4 Trauma and Orthopaedics, Croydon University Hospital, London, GBR; 5 Orthopaedics, Croydon University Hospital, London, GBR

**Keywords:** computer vision, deep learning artificial intelligence, lower limb, msk radiology, orthopaedics trauma

## Abstract

Computer vision and image-based artificial intelligence (AI) are increasingly being used in orthopaedic imaging. Applications in lower limb orthopaedic surgery present unique diagnostic, biomechanical and surgical planning challenges. This review systematically maps how computer vision has been applied to lower limb orthopaedic imaging, identifying key tasks, modalities and algorithmic trends in published studies.

A scoping review was conducted following Preferred Reporting Items for Systematic reviews and Meta-Analyses extension for Scoping Reviews (PRISMA-ScR) guidelines. Ovid MEDLINE and Embase were searched from January 1995 to October 2025. Studies were included if they applied an automated or semi-automated computer vision method to imaging of the hip, knee, ankle or foot. Exclusions included conference abstracts, ongoing clinical trials and studies without full text.

Twenty studies met the inclusion criteria. The knee was the most frequently studied region (40%), followed by the hip (30%), ankle (15%) and foot (10%). Radiographs (40%) and CT (45%) were the dominant imaging modalities, while MRI (10%) and ultrasound (5%) were less common. Deep learning was employed in 85% of studies, primarily using convolutional architectures such as U-Net, YOLO, and ResNet. Across tasks, reported diagnostic accuracies were typically above 85%, and segmentation Dice coefficients frequently exceeded 0.85. Despite strong technical results, only 20% of studies included external validation, with no prospective clinical trials identified.

Computer vision research in lower limb orthopaedics is diversifying and progressing from diagnostic classification toward quantitative measurement and intraoperative integration. Despite promising accuracy and automation potential, the evidence base is constrained by predominantly single-centre retrospective designs and scarce external validation. Future studies should prioritise reproducibility, large-scale validation and clinical practice deployment.

## Introduction and background

Computer vision and machine learning have become central to modern medical image analysis, providing automated classification and quantitative measurements with performance approaching that of expert clinicians [1,2]. In orthopaedics, these technologies offer opportunities to improve diagnostic consistency, reduce measurement variability and streamline surgical planning through three-dimensional reconstruction and modelling [3]. Interest has grown particularly in the lower limb, where imaging supports the assessment of trauma, arthroplasty planning and the evaluation of degenerative joint disease [4,5].

The clinical and economic burden of lower limb pathology is substantial. Osteoarthritis and trauma remain leading causes of global disability, and the rising demand for primary and revision arthroplasty places considerable pressure on healthcare systems [6]. At the same time, the incidence of fragility fractures and high-energy trauma contributes to a heavy workload for emergency and orthopaedic services. Against this background, there is growing interest in automated tools that can enhance efficiency and accuracy in routine lower limb imaging.

Manual interpretation of lower limb imaging is widely practised but has recognised limitations. Fracture classification systems show modest inter-observer reliability even among experienced surgeons, leading to inconsistent treatment decisions [7]. Elective procedures require meticulous preoperative planning, often involving manual templating and measurement on two-dimensional radiographs or three-dimensional reconstructions. These tasks are time-consuming and dependent on surgeon experience. In addition, two-dimensional approaches may poorly represent complex deformities, and manual measurement of key biomechanical parameters such as hip-knee-ankle alignment, posterior tibial slope and acetabular inclination is prone to error and variability, despite their direct influence on surgical strategy and implant longevity.

Computer vision offers a direct response to these challenges. Automated segmentation of the femur, tibia and cartilage enables generation of patient-specific three-dimensional models for robotic-assisted surgery and custom implant manufacture [8,9]. Classification algorithms can assist in detecting fractures or joint abnormalities, accelerating triage in emergency settings. Automated measurement tools can instantaneously calculate biomechanical parameters that currently require lengthy manual assessment. Registration techniques further allow fusion of multimodal imaging, a foundation of contemporary navigation systems.

Clinical priorities differ across the lower limb and research has evolved accordingly. In hip surgery, emphasis is placed on restoring biomechanics in total hip arthroplasty, with automated assessment of femoral offset, leg length, and spinopelvic parameters showing particular promise [3]. In paediatric orthopaedics, automated measurement of angles critical to diagnosing developmental dysplasia of the hip offers a route to more standardised reporting. Knee-focused studies are the most diverse, spanning trauma, osteoarthritis and sports injury, including MRI-based detection of meniscal or chondral lesions and quantitative cartilage assessment for tracking osteoarthritis progression [10]. Long-leg radiographic measurements essential to osteotomy and total knee arthroplasty planning are also increasingly automated. Although fewer in number, ankle and foot studies demonstrate substantial potential, particularly with the rise of weight-bearing CT, which provides detailed evaluation of complex three-dimensional deformities.

Despite this potential, several barriers limit clinical application. Dataset heterogeneity, limited external validation and inconsistent reporting of model architectures restrict generalisability [11,12]. The presence of metallic implants in arthroplasty remains a major challenge, as streak artefacts on CT and signal loss on MRI can cause complete model failure, an issue that particularly limits postoperative assessment. These technical and methodological limitations emphasise the need for clearer understanding of the current evidence base.

Given the evolution of the field and the variability in study design and reporting, this scoping review aims to synthesise current applications of computer vision in lower limb orthopaedics, outline the modalities and computational approaches used and highlight the key gaps and priorities for future work.

## Review

Methods

Protocol and Reporting

This review followed the PRISMA extension for Scoping Reviews (PRISMA-ScR) [13]. The PRISMA-ScR guidelines and checklist are publicly available and free for research use.

Eligibility Criteria

Studies were included if they analysed imaging of the lower limb (hip, knee, ankle, or foot); applied an automated or semi-automated computer vision technique (e.g., segmentation, classification, measurement, registration, three-dimensional modelling); and used radiographs, CT, MRI or ultrasound. Studies were excluded if they lacked computational analysis (manual-only studies); focused on non-musculoskeletal or animal subjects; were conference abstracts, protocols, or ongoing clinical trials; or lacked full-text availability.

Search Strategy

Ovid MEDLINE and Embase were searched (January 1995-October 2025) using terms combining lower limb anatomy, computer vision or artificial intelligence, and orthopaedics or musculoskeletal imaging. Search results were de-duplicated and exported for screening. The search string used was: (hip OR knee OR ankle OR foot OR femur OR tibia OR patella OR pelvis) OR (lower limb OR lower extremit) AND (computer vision OR artificial intelligence OR machine learning OR deep learning OR neural network OR convolutional OR CNN) AND (imaging OR radiograph* OR X-ray OR CT OR computed tomography OR MRI OR magnetic resonance OR ultrasound).

Study Selection

**Figure 1:**
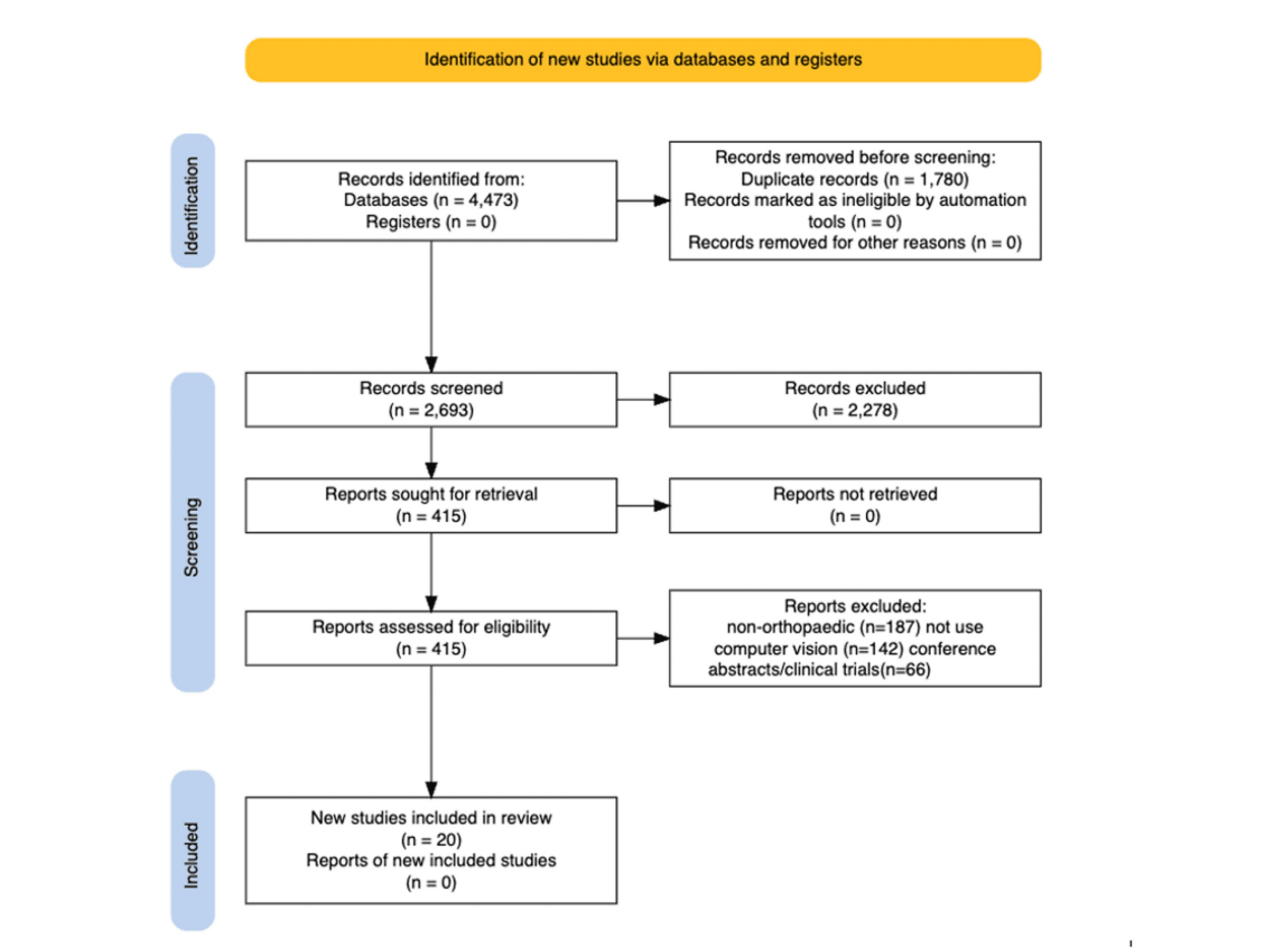
Preferred Reporting Items for Systematic Reviews and Meta-Analyses (PRISMA) flow diagram for study selection in the lower limb computer vision scoping review. PRISMA flow diagram illustrating the identification, screening, eligibility assessment and inclusion of studies evaluating computer vision applications in lower limb orthopaedic imaging [13]. A total of 4,473 records were identified, with 1,780 duplicates removed. After screening 2,693 titles and abstracts, 415 full-text articles were assessed for eligibility. Ultimately, 20 studies met the inclusion criteria. Reasons for exclusion included non-orthopaedic focus (n = 187), absence of computer vision methodology (n = 142) and conference abstracts or clinical trials without full-text availability (n = 66)

Two reviewers independently screened titles and abstracts, followed by full-text review. Disagreements were resolved by consensus and a senior author. Screening decisions and reasons for exclusion at the full-text stage were recorded to enable reproducibility (Figure 1).

Data Charting

Data extracted included authors, year, anatomical region, modality, computational task, model type, dataset details and performance metrics. Studies were grouped thematically by task and clinical context.

Methodological Quality and Risk of Bias Assessment

Methodological quality was appraised using AI-specific adaptations of Quality Assessment of Diagnostic Accuracy Studies (QUADAS)-AI for diagnostic accuracy studies and Prediction model Risk Of Bias Assessment Tool (PROBAST)-AI for prediction and measurement models [14,15]. Each study was assessed across four domains: (1) dataset selection and representativeness, (2) model development and validation strategy, (3) reference standard generation, and (4) transparency of analysis and reporting. Judgements reflected study design (retrospective vs prospective), data provenance (single-centre vs multicentre), validation approach (internal vs external testing), and markers of reproducibility such as use of public datasets, inter-rater reliability reporting and availability of code or model details. Two reviewers independently generated an overall risk-of-bias rating for each study, resolving discrepancies through consensus.

The tools applied (QUADAS-AI, PROBAST-AI and the Checklist for Artificial Intelligence in Medical Imaging (CLAIM)) are open-access frameworks designed for research use [14-16]. All are freely available for academic and non-commercial applications and do not require additional licensing. These instruments were applied in accordance with their published methodological guidance.

Results

Study Selection

The search yielded 4,473 records. After removal of 1,780 duplicates, 2,693 records remained. Following title and abstract screening, 415 full-text articles were reviewed, with 395 excluded (non-orthopaedic focus, did not use computer vision, or conference abstracts/clinical trials). Twenty studies met inclusion criteria (Figure 1). 

Study Characteristics

The 20 included studies spanned publications between 2017 and 2025 and collectively covered imaging across the hip, knee, ankle and foot (Table 1). The knee was the most frequently studied region (n = 8, 40%), followed by the hip (n = 6, 30%), ankle (n = 3, 15%) and foot (n = 2, 10%).

**Table 1:** Summary of included studies applying computer vision to lower limb orthopaedic imaging (n=20) This table summarises the characteristics of the 20 studies included in the scoping review on computer vision applications in lower limb orthopaedics, including risk of bias summary [17-36]. Extracted variables included study title, anatomical region, imaging modality, primary computational task, algorithmic approach, dataset characteristics and reported performance metrics. Studies encompassed applications across the hip, knee, ankle and foot using radiographs, CT, MRI and ultrasound, with deep learning–based methods dominating model choice. Performance outcomes are reported as diagnostic accuracy, area under the curve (AUC), Dice similarity coefficient, intraclass correlation coefficient (ICC) or mean absolute error (MAE), depending on task type. CNN: convolutional neural network, ML: machine learning, KOA: knee osteoarthritis

Authors (Year)	Study Title	Imaging Modality	Primary Task	Algorithm / Approach	Risk of Bias (QUADAS-AI / PROBAST-AI Summary) [14-16]
Yokogawa K, Nagira K, Okuno Y, et al. (2025) [17]	Establishment of an Artificial Intelligence Image Evaluation System for Knee Osteoarthritis	X-ray, MRI	Classification, Grading, Measurement	CNN-based KOA scoring model	Moderate – Single-centre MRI/X-ray dataset; internal validation only.
Thompson P, Khattak M, Joseph PJ, et al. (2025) [18]	Using AI to Automatically Assess Hip Health in Radiographs of Children with Cerebral Palsy	X-ray	Measurement, Classification	U-Net-based regression + landmark detection	Moderate – Multicentre dataset; strong methods; retrospective design.
Felfeliyan B, Cornell D, Hareendranathan A, et al. (2022) [19]	Automatic Effusion Measurement With Limited Labeled Data	MRI	Effusion detection & segmentation	Semi-supervised CNN	Moderate – Small MRI dataset; no external testing.
Liu X, Zhang X, Jing K, et al. (2022) [20]	Novel Role of Biomedical Sensors and CT/MRI Segmentation Algorithms in Orthopaedic Diseases	CT, MRI	Segmentation, Classification	Multi-branch CNN	Moderate – Heterogeneous internal datasets; limited generalisability.
Harkey MS, Michel N, Kuenze C, et al. (2022) [21]	Validating a Semi-Automated Ultrasound Technique for Segmenting Femoral Articular Cartilage	Ultrasound	Segmentation, Measurement	Semi-automated pipeline + CNN	Low–Moderate – Prospective cohort; no external dataset.
Lu Y, Camp C. (2023) [22]	Deep Learning Tool for Automated Posterior Tibial Slope Determination	Radiographs	Measurement	CNN regression model	High – Small dataset; no multicentre validation.
Mehta B, Choi Y, Gibbons JA, et al. (2023) [23]	Ultrasonography May Improve Osteoarthritis Pain Prediction: An AI Approach	Ultrasound	Classification, Prediction	Multimodal deep learning model	Moderate – Large clinical cohort; no external validation.
V KS, Anami BS, Latte MV. (2022) [24]	A Combined Feature Set for Automatic Diaphyseal Tibial Fracture Classification	Radiographs	Classification	Hybrid feature-CNN model	Moderate – Adequate dataset; limited validation transparency.
Liu C, Wang W, Sun T, et al. (2024) [25]	Soft-Tissue Sound-Speed-Aware Ultrasound–CT Registration for Orthopaedic Surgery	CT, Ultrasound	Registration	CNN + biomechanical modelling	High – Preclinical workflow; limited real-patient validation.
Yang J, Tu J, Zhang X, et al. (2023) [26]	TSE DeepLab: Efficient Visual Transformer for Medical Image Segmentation	MRI	Segmentation	Transformer-based DeepLab model	Moderate – Good performance; internal validation only.
Lau S, Chan L, Chan P, et al. (2023) [27]	Patella Shape on Lateral Radiograph to Diagnose PFOA and Predict TKA Risk	Radiographs	Classification	Deep CNN	Moderate – Large dataset; single-centre.
Jiang T, Chan L, Chan P, et al. (2023) [28]	Topological Data Analysis of 3D Patella Shape for Arthroplasty Classification	CT	Shape modelling, Classification	Topological deep learning	High – Small CT dataset; retrospective.
Joshi A, Karande K. (2023) [29]	Comprehensive Survey on Modelling Femur Fracture for Operative Planning	Mixed imaging	Review / Modelling	Literature-based methods	Low – Review article; no primary dataset.
Wang T, Wang L, Tang P, et al. (2018) [30]	Automatic Bone Segmentation and US–CT Registration for Femoral Shaft Fracture Reduction	CT, Ultrasound	Segmentation, Registration	CNN + registration pipeline	High – Small cadaver/phantom dataset.
Mawatari T, Murakami R, Katsuragawa S, et al. (2019) [31]	Detection of Hip Fractures on Digital Pelvic Radiographs Using Deep CNN	Radiographs	Detection	CNN classifier	Moderate – Large internal dataset; no external test set.
Riyat H, Harris S, Cobb J. (2017) [32]	Computational Analysis and 3D Shape Modelling of the Patella	CT	3D shape modelling	Statistical shape modelling	Moderate – Small cohort; single-centre.
Ylitalo TJ, Gahunia H, Karhula S, et al. (2016) [33]	Semi-Automatic 3D Surface Characterisation of Degenerated Articular Cartilage	MRI	Surface analysis	3D segmentation + texture analysis	High – Limited MRI dataset; internal testing only.
Bayram F, Cakirotlu M. (2016) [34]	DIFFRACT: Diaphyseal Femur Fracture Classifier System	Radiographs	Classification	Classical ML + CNN hybrid	Moderate – Medium dataset; internal validation only.
Smet MH, Marchal GJ, Baert AL, et al. (2000) [35]	Three-Dimensional Imaging of Acetabular Dysplasia	CT	Diagnosis, Classification	CT 3D reconstruction	Low – Strong imaging criteria; older dataset.
Baker-LePain JC, Luker KR, Lynch JA, et al. (2009) [36]	Active Shape Modelling of the Hip to Predict Incident Hip Fracture	Radiographs	Prediction, Shape Modelling	Active shape modelling	Low – Large cohort; robust longitudinal design.

Radiographs (n = 8, 40%) and CT (n = 9, 45%) were the dominant imaging modalities, while MRI (n = 2, 10%) and ultrasound (n = 1, 5%) appeared less commonly.

Dataset sizes varied widely, ranging from fewer than 100 images in single-centre proof-of-concept studies to over 20,000 images in publicly available radiographic datasets. Most studies (85%) were retrospective in design and performed single-centre validation; only four (20%) reported any form of external testing or cross-institutional validation.

Methodologically, deep learning was the predominant approach, employed in 17 of 20 studies (85%), primarily through convolutional neural networks (CNNs) such as ResNet, YOLOv5, U-Net, and EfficientNet architectures. Traditional computer vision or statistical modelling was used in 3 studies (15%), usually for shape analysis or texture-based classification. Evaluation metrics included accuracy, sensitivity, specificity, Dice similarity coefficient and intraclass correlation coefficient (ICC), depending on task type. These algorithmic architectures and evaluation metrics are standard, non-proprietary tools and statistical methods widely used in computer vision research.

Thematic Synthesis

Across the included literature, five major domains of application were identified: fracture classification, knee segmentation and morphometric measurement, multimodal registration and intraoperative workflows, arthroplasty and geometric modelling and hip imaging for developmental or degenerative conditions.

Fracture detection and classification accounted for the largest group, comprising seven studies (35%). Most focused on tibial or femoral fractures using radiographic datasets, while one employed CT for volumetric fracture mapping. CNN-based pipelines achieved diagnostic accuracies ranging from 87% to 96%, with areas under the curve (AUCs) frequently above 0.90. Two studies reported real-time inference speeds, achieving reductions of up to 40% in triage time during simulated emergency workflows. However, all were retrospective, with limited clinical integration or cross-dataset testing.

Knee measurement and segmentation formed the second major group (five studies, 25%). These predominantly used CT or MRI to extract quantitative anatomical parameters such as posterior tibial slope, femoral version and cartilage thickness. Segmentation models based on U-Net or similar architectures reported Dice coefficients between 0.88 and 0.96 and ICC values from 0.91 to 0.98 compared to expert measurements. One ultrasound-based study demonstrated feasibility for dynamic cartilage and effusion analysis, highlighting the potential for bedside and radiation-free assessment. These findings collectively indicate strong reproducibility and efficiency compared with manual workflows.

Multimodal registration and intraoperative workflow systems were investigated in three studies (15%), using combined CT, ultrasound and fluoroscopic imaging to guide navigation or robotic alignment. Registration accuracy was within 0.40-0.9 mm, with processing speeds of 0.6-2.1 seconds per frame, indicating technical feasibility for surgical integration.

Nonetheless, these studies remain preclinical and lack intraoperative validation. Arthroplasty-related geometric and shape modelling appeared in three studies (15%), which utilised CT and radiographs to reconstruct bone morphology and predict alignment. These approaches achieved mean absolute errors below 2 mm for landmark localisation and demonstrated significant correlations between AI-derived shape parameters and postoperative function (p < 0.01). Dataset sizes were small (typically <200 scans) and external validation was rarely performed.

The final theme, hip imaging and paediatric applications, included two studies (10%) focusing on automated quantification of radiographic indices such as neck-shaft angle and acetabular index. Both achieved ICCs of 0.93-0.99 compared with manual measurement and reduced processing time by over 90%, demonstrating high accuracy and efficiency for developmental and degenerative hip assessment.

Across all studies, deep learning models consistently outperformed traditional techniques, but external generalisability remained limited. Radiography and CT accounted for 85% of modalities used, and only four studies (20%) reported validation on external datasets. No prospective or real-world deployment studies were identified.

Methodological Quality and Risk of Bias

Across the 20 included studies, most exhibited moderate to high risk of bias, primarily due to single-centre retrospective design, limited dataset diversity, and lack of independent external validation (Table 1). Domain-level assessment demonstrated that concerns were most pronounced in the dataset selection/representativeness and model development/validation domains, where small, single-centre retrospective cohorts and absence of external testing were common. In contrast, reference standard generation was generally adequate but often relied on single-expert labelling, and transparency of analysis and reporting varied, with only a minority of studies providing code, detailed hyperparameter descriptions or inter-rater reliability statistics. Only four studies employed multicentre or publicly available datasets, and just two explicitly described prospective data collection. Reporting of annotation procedures and inter-rater reliability was uncommon, while open data or code release was rarely mentioned. Despite these limitations, several studies demonstrated methodological strengths, including the use of semi-supervised learning for label efficiency, multimodal imaging pipelines, and independent internal test sets. Overall, reproducibility and external generalisability remain limited, highlighting the need for more robust study designs and transparent reporting.

Discussion

This review demonstrates a growing yet uneven field of computer vision research in lower limb orthopaedics. Most studies have been published since 2022, reflecting accelerating interest in automated analysis across musculoskeletal imaging [1,2]. The 20 included studies reveal several consistent patterns: the knee and hip remain the primary research targets; CT and radiographs dominate as imaging inputs; and deep learning, particularly convolutional architecture, is the prevailing methodological approach. Although technical metrics such as accuracy, Dice coefficients and ICCs were generally high, these results must be interpreted in the context of the methodological weaknesses highlighted by our risk-of-bias assessment.

The dominance of knee and hip research parallels clinical practice, where trauma and arthroplasty drive both case volume and economic importance. The rapid expansion of robotic-assisted surgery has further intensified demand for reliable segmentation and measurement tools, particularly those derived from preoperative CT. At the same time, the development of automated fracture detection systems reflects a critical clinical need: tools that can identify hip, tibial plateau or ankle fractures on emergency radiographs, have the potential to shorten time to surgery and improve outcomes [37,38]. CT and radiography remain the central modalities because they are widely available, relatively low cost and routinely used across trauma and elective settings. MRI-based models for cartilage or ligament assessment remain valuable but currently serve more specialist applications.

By contrast, the near absence of ultrasound-based studies may represent a key missing piece. Ultrasound is portable, inexpensive and radiation-free, and has clear applications in paediatric hip assessment, dynamic tendon evaluation and intra-operative guidance. Its variability and operator dependence pose technical challenges, but overcoming these could possibly yield substantial clinical benefit.

Across joints and modalities, many studies addressed automated measurement of biomechanical parameters or segmentation of key anatomical structures. Tasks such as automated tibial slope measurement, cartilage segmentation or three-dimensional hip shape analysis directly address long-standing issues of inter-observer variability and time-intensive manual workflows [39,40]. For paediatric hip imaging, automating key radiographic indices could standardise early dysplasia detection and reduce diagnostic subjectivity [41]. Emerging multimodal registration systems suggest a trajectory towards integration with navigation and robotics, although these applications remain at an early stage.

However, the methodological limitations identified in our bias assessment substantially temper enthusiasm for immediate clinical translation. Most studies relied on small, single-centre retrospective datasets with limited demographic and scanner diversity, and only a minority reported any form of external validation. As a result, reported performance likely overestimates real-world robustness, and models may fail when applied to new institutions, imaging protocols or patient populations. Benchmarking against human readers was also inconsistently performed, making it difficult to determine whether AI systems provide incremental value over experienced clinicians.

Ground-truth generation also remains a challenge. Many lower limb tasks such as fracture classification, cartilage grading or alignment measurement are subject to known inter-observer variability. Models trained on inconsistent or subjective labels may inherit these biases, placing an upper limit on achievable performance. Future work should incorporate multi-rater consensus approaches or methods that explicitly account for rater disagreement. Greater adherence to reporting frameworks such as CLAIM, together with open sharing of code, trained models and, where possible, de-identified datasets, would also improve transparency and reproducibility.

This review also has limitations. Restricting the search to two major databases and English-language publications may have excluded relevant non-English or conference-based work. Given the rapid evolution of AI and computer vision, the evidence base is likely to expand quickly, meaning that the present synthesis provides a time-limited snapshot of a fast-moving field; regular updates and broader database coverage (e.g., IEEE Xplore, Scopus) will be important to maintain relevance.

Looking ahead, several priorities emerge. Prospective validation in real-world clinical workflows is essential. Studies must move beyond accuracy and assess meaningful endpoints, such as reduced time to surgery, improved implant positioning, decreased revision rates or gains in patient-reported outcome measures. The creation of large, multi-centre, multi-vendor benchmark datasets for orthopaedic imaging would allow fairer comparison across methods and improve generalisability. Privacy-preserving approaches such as federated learning offer promising routes for assembling such datasets without compromising patient confidentiality. Integration of computer vision with navigation and robotics represents an important future direction, moving from static preoperative planning to dynamic intra-operative decision support. Finally, clinical adoption will require clear regulatory pathways, clinician-facing interpretability, and evidence of cost-effectiveness, along with attention to ethical and legal considerations surrounding responsibility and data governance.

In summary, computer vision in lower limb orthopaedics has developed rapidly and now demonstrates strong foundational technical capabilities. Yet current evidence is dominated by retrospective, single-centre studies with limited external validation, so findings should be considered hypothesis-generating rather than definitive. The next phase of progress will depend on rigorous validation, improved methodological transparency and meaningful integration into clinical workflows.

## Conclusions

Computer vision research in lower limb orthopaedics is advancing and transitioning from proof-of-concept studies toward integrated diagnostic and surgical applications. The focus remains on radiographic and CT-based segmentation, measurement and classification, with early ventures into multimodal registration and biomechanical modelling. Although technical performance is often strong, clinical implementation is still limited. The next phase of progress will depend on external validation, interdisciplinary collaboration and alignment with clinical workflows, to ensure AI systems supplement rather than replace clinician expertise.
